# Detection of Minimal Residual Disease by Flow Cytometry for Patients with Multiple Myeloma Submitted to Autologous Hematopoietic Stem Cell Transplantation

**DOI:** 10.1155/2013/847672

**Published:** 2013-06-20

**Authors:** Suzane Dal Bó, Annelise Pezzi, Bruna Amorin, Vanessa Valim, Rosane Isabel Bittencourt, Lucia Silla

**Affiliations:** ^1^Unit of Hematology, Clinical Pathology Service, Hospital de Clínicas de Porto Alegre, 90035-903 Porto Alegre, RS, Brazil; ^2^Laboratory of Cell Culture and Molecular Analysis of Hematopoietic Cells, Hospital de Clínicas de Porto Alegre, 90035-903 Porto Alegre, RS, Brazil; ^3^Hematology and Bone Marrow Transplantation, Hospital de Clínicas de Porto Alegre, 90035-903 Porto Alegre, RS, Brazil

## Abstract

The treatment strategy in multiple myeloma (MM) is to get complete remission followed by high-dose chemotherapy and autologous Hematopoietic Stem Cell Transplantation (HSCT). Neoplastic Plasma Cells (NPCs) are CD45^−/dim^, CD38^+high^, CD138^+^, CD19^−^, and  CD56^+high^ in most cases. The description of this immunophenotype is of major importance as it leads to the correct identification of minimal residual disease (MRD). Samples from 44 Patients were analyzed prospectively in this study. We analyzed if the presence of MRD at three months after HSCT was predictive of relapse or death. There were 40 evaluable patients of whom 16/40 patients had MRD at three moths after HSCT and there were none in cytological relapse. The mean overall survival (OS) was 34 months and disease-free survival (RFS) was 28 months after HSCT. There was no significant difference in the log rank analysis comparing OS and the presence of MRD (*P* = 0,611) and RFS (*P* = 0,3106). Here, we demonstrate that three color flow cytometry (FCM) is more sensitive for MDR evaluation than cytological analyzes. However, based in our data we can not affirm that MRD is a good predictor of MM relapse or death. In conclusion, our results could be attributed to a short followup, small sample size, and over most to the inability of a three-color FCM to detect the NPC population.

## 1. Introduction

 Multiple myeloma (MM) is a malignant disease characterized by an increase in the number of clonal plasma cells in the bone marrow (BM) and the presence of monoclonal protein, the M-protein, usually IgG or IgA, in blood, urine, or both [1]. Clinical signs are different combinations of bone marrow plasma cell infiltration with or without impaired hematopoiesis [2]; production of monoclonal immunoglobulin with decrease in the production of normal, polyclonal gammaglobulins; osteolytic lesions [3, 4], hypercalcemia, and renal failure. Since the clinical picture is heterogeneous, diagnostic criteria are mandatory in routine clinical practice being the Durie and Salmon staging criteria the gold standard to diagnose and stage MM. These criteria combine hemoglobin 2 g/dL below the normal level for the laboratory or if the haemoglobin falls to 10 g/dL, a serum calcium level >0.25 mmol/L, the serum creatinine >173 mmol/L, M-protein in serum >30 g/L, and bone involvement [5, 6]. A finding of 10% or more plasma cells in bone marrow aspirate (BMA) is one of the three major criteria for the diagnosis of MM. For risk stratification, apart from the Durie and Salmon criteria, the International Myeloma Foundation has recently recommended the International Staging System (ISS), a new set of criteria based on the values of β_2_ microglobulin and serum albumin [7]. Because of the heterogeneous distribution in the BM, the variation of plasma cell percentage is not a criterion to evaluate response to treatment, but the detection of less than 5% of plasma cells, associated with the disappearance of other signs and symptoms of the disease, is generally accepted as complete remission [8].

**Figure 1:**
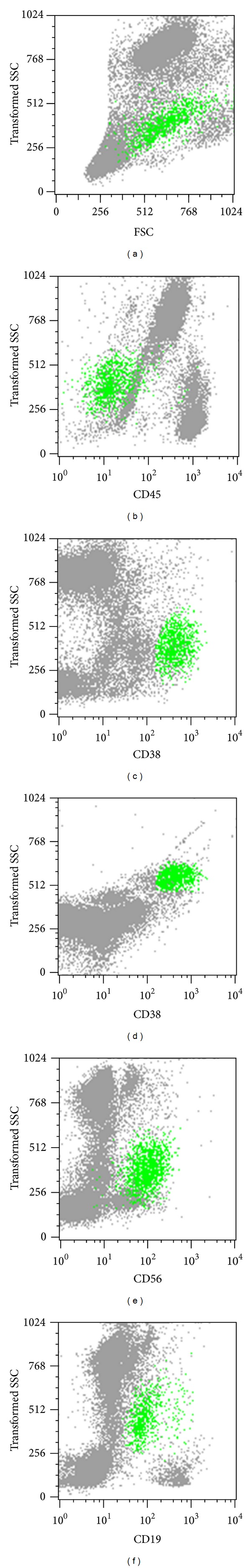
Immunophenotypic characteristic plasma cell (painted in green). (a) SSC × FSC characteristics of normal PC and neoplastic PC, (b) CD45^−/dim^ characteristics of neoplastic PC, (c) CD38^+high^ characteristics of normal and neoplastic PC, (d) CD138 × CD38 characteristics of normal PC and neoplastic plasma cell, (e) CD56^+high^ characteristics of neoplastic PC, (f) CD19 negative characteristics of neoplastic PC. *TSSC (transformed SSC—Paint a Gate).

Plasma cells are characterized by the presence of cytoplasmic immunoglobulin and, on the cell surface, CD38 and CD138 antigens [8–12]. The CD38 is widely expressed in the hematopoietic lineage; flow cytometry (FCM) has shown that the intensity of CD38 fluorescence in plasma cells is much higher than in the other hematopoietic cells, and this strong reactivity was converted into a specific marker for plasma cells. CD138 (syndecan-1) is a specific marker, both for normal and neoplastic plasma cells since it is not found in the other hematopoietic cells [13–15]. The CD38 and CD138 combination in flow cytometry is widely used to characterize both normal and neoplastic plasma cells [9–11, 16–18]. At the same time, neoplastic plasma cells lose the CD19, CD20, and CD22 markers in about 85% of the patients with MM [14, 15, 19] and there is little or no expression of CD45 in about 90% of the cases [20]. Adhesion molecules apparently involved in the pathogenesis of MM, such as the CD56 [8], are found in about 70% [19] of the patients with MM and disappear in advanced stages of the disease [21, 22]. Another well-established characteristic of plasma cells is their size properties (f*orward scatter*—FSC) and internal complexity (s*ide scatter*—SSC), which are revealed in the analysis by flow cytometry [8]. The combination of these characteristics by multiparametric immunophenotyping allows us to study the coexpression of these molecules on the cell surface, to detect malignant plasma cells, and to differentiate MM from other monoclonal gammopathies. In summary, differently from other normal plasma cells (CD19^+^, CD45^+^, CD38^+high^, CD138^+^, and CD56^−^), neoplastic plasma cells are CD45^−/dim^, CD38^+high^, CD138^+^, CD19^−^, and CD56^+high^ (Figure 1) in most cases. The identification of this immunophenotype has substantial value in the distinction between MM and monoclonal gammopathy of undetermined significance (MGUS), as well as in the assessment of minimal residual disease (MRD) [8, 16, 19, 23–25].

MRD may be defined as the presence of neoplastic cells in patients that are clinically in complete remission (CR), detected by more sensitive methods than light microscopy, such as FCM [10, 17] or polymerase chain reaction (PCR) [26, 27]. Methods to detect residual tumor cells or early relapses without clinical signs have been widely used in an attempt to initiate adequate therapy for MRD as early as possible after transplantation [28]. The role of the presence and amount of MRD is well established in chronic myeloid leukemia (CML) [29] and in acute lymphoid leukemia (ALL) in children [30]. In both, the amount of MRD measured using quantitative PCR for CML e LLA and FCM for ALL is associated with disease relapse.

 The best treatment strategy in MM is to get CR followed by high-dose chemotherapy and autologous Hematopoietic Stem Cell Transplantation (HSCT) [31]. Thirty-one to 51% of these patients achieve CR [32], and this is followed by higher rates of disease-free or progression-free survival when compared with patients that do not achieve CR [33]. In this scenario, however, of the patients in CR only a few achieve molecular remission eventually relapsing as consequence of residual disease [34]. The International Myeloma Working Group has defined stringent complete response (sCR), CR, very good partial remission (VGPR), and partial remission (PR) as a tool to compare and analyze treatment results [35]. Briefly, sCR is defined as CR plus normal free light chains (FLC ratio) and absence of phenotypically aberrant plasma cells-PC in bone marrow with a minimum of 3000 total PC analyzed by multiparametric flow cytometry (with >4 colors) CR is defined as absence of M component (serum and urine) and ≤5% plasma cells in the BM; VGPR is serum and urine M component detectable by immunofixation but not on electrophoresis or 90% or greater reduction in serum M-protein plus urine M-protein level <100 mg per 24 h and PR ≥50% reduction of serum M-protein and reduction in 24 h urinary M-protein by ≥90% on to <200 mg per 24 h.

Molecular [9, 10, 18, 36, 37] or FCM [9, 10, 17, 24, 26, 36] methods have been used to detect MRD in MM, but its clinical importance is still being evaluated since most studies included only a small number of patients [10, 18, 24].

 This prospective study evaluated MRD detection using FCM in patients with MM submitted to high-dose chemotherapy followed by autologous Hematopoietic Stem Cell Transplantation (HSCT), at the Hematology and Bone Marrow Transplantation Unit at Hospital de Clínicas de Porto Alegre, Rio Grande do Sul, Brazil. 

## 2. Material and Methods

### 2.1. Patients and Treatment

Patients with MM who achieved CR, VGPR, or PR to high-dose chemotherapy (MEL 200—melphalan 100 mg/m^2^/day [D-3] and melphalan 100 mg/m^2^/day [D-2]) followed by autologous HSCT between December 2005 and May 2009 were included in this study. We evaluated the patients at 3 months after HSCT and the laboratory reevaluation was performed and bone marrow was collected for cytological and immunophenotypic studies. This study was approved by the Ethics Committee of HCPA. Written informed consent was obtained from every participant and the data were analyzed anonymously according to Declaration of Helsinki for human studies. 

### 2.2. Immunophenotypic Studies

The bone marrow samples were prepared and analyzed within 24 hours. Each test tube received 100 *µ*L of BM, which corresponded to about 10^6^ leukocytes; samples were incubated with 5 *µ*L of each monoclonal antibody marked with fluorochromes (FITC, PE, and PECY5/PERCP), according to the panel below, and incubated for 15 minutes in the dark; the lysis was performed using Facslyse (BD Dickinson, San Jose, CA, USA) for 15 minutes, centrifuged, washed twice with PBS (phosphate buffer saline), and resuspended with PBS plus paraformaldehyde. The cells were acquired in a 3-color FacsCalibur BD Flow Cytometer 10 (Becton Dickinson, San Jose, CA). 

The monoclonal antibodies used were CD38, CD138, CD19, CD56, and CD45 in the following combination: CD45/CD138/CD38; CD19/CD138/CD38; CD38/CD56/CD138; and CD45/CD3. Samples were acquired twice: first, 20000 events, and second, using the Gates acquisition system, 50000 to 450000 events were acquired in the CD38 gate. The acquisition software used was Cellquest BD (Becton Dickinson, San Jose, CA, USA). In the gate acquisitions, the total number of events acquired in the cytometer was recorded for the final calculation of the number of cells with phenotypes of interest. The analyses were performed using the Paint-A-Gate PRO software (Becton Dickinson, San Jose, CA, USA). Neoplastic plasma cells were defined as CD45^−/dim⁡^CD138^+^, CD38^+high^, and CD19^−^ CD56^+/−^. CD3 was utilized to exclude contaminating events and to detect peripheral blood in the sample. The analytical strategy was to choose positive events for CD38 and CD138 and to check the expression of CD45, CD56, and CD19. A minimum of 50 positive events, considering the sum of the three tubes under analysis, was required to ensure analysis. MRD was defined as the presence of neoplastic plasma cells higher than 0.01% of the sample as described in previous studies [8, 18, 25].

Cytological examination was done in BM cells smear stained with MayGrunwald Giemsa including at least 500 nucleated cells. 

The end points of the study were to evaluate a possible relationship between the presence of MRD, relapse, or death in relapse during the period of the study. 

### 2.3. Statistical Methods

A descriptive analysis of the data was accomplished through median for the quantitative variables, while the categorical variables were represented through frequency and percentile. For evaluation of the overall survival (OS) and disease-free survival (DFS) Kaplan-Meier was utilized, comparing the groups through the log rank test. The data was analyzed in SPSS 12.0 and the value of adopted alpha was 5%. 

## 3. Results

Samples from 44 patients (21 men) were analyzed prospectively in this study. Median age at the time of HSCT was 55 years [percentile-25 (52, 25 years) and 75 (64 years)] (Table 1). 

**Figure 2:**
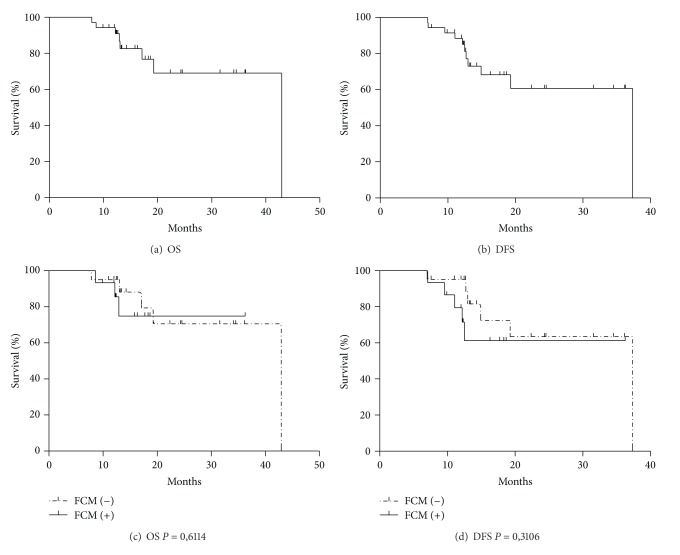
(a) Overall survival (OS), (b) disease-free survival (DFS), (c) overall survival curves according to the DRM evaluation, (d) disease-free survival curves according to the DRM evaluation.

We analyzed if the presence of MRD at three months after HSCT was predictive of relapse or death. There were 40 evaluable patients of whom 16/40 patients had MRD at three months after HSCT and there were none in cytological relapse (Table 2). The mean overall survival (OS) was 34 months and disease-free survival (DFS) was 28 months (Kaplan-Meier curve) after HSCT. There was no significant difference in the log rank analysis comparing OS and the presence of MRD (*P* = 0,611) and relapse-free survival (RFS) (*P* = 0,3106) (Figure 2). Although not all the patients were evaluated for MRD in all time points, our results showed FCM MDR evaluation to be more sensitive than BM, in none of these points; however, they were MRD predictive of relapse or death of MM. 

## 4. Discussion

 Pérez-Persona et al. [38] demonstrated that the ratio between the proportion of abnormal and normal plasma cell as identified by FCM significantly correlates with risk for disease progression. A better long-term outcome was observed in patients with a low level of MM plasma cells as detected by FCM prior to autologous HSCT [24]. Early reappearance of MM plasma cells after high dose chemotherapy was related to a shorter PFS [10, 33]. Paiva et al. [39] showed in a large number of uniformly treated MM, 297 patients, that FCM MRD status at day 100 after autologous HSCT was the most relevant prognostic factor for MM. 

 Here we showed that in spite of being significantly more sensitive than cytology to detect MRD in MM, FCM—measured by the identification of plasma cells CD45^−/dim^ CD138^+^, CD38^+high^, and CD19^−^ CD56^+^ [13–15, 23]—did not predict for relapse or death from MM in our group of patients. 

 The correct quantification of plasma cells in bone marrow is fundamental for the diagnosis of MM [35]. Multiparametric flow cytometry is a method to monitor minimal residual disease and to evaluate treatment results [16]. The use of this method will eventually become more frequent in this context and, therefore, demand approaches that define specificity and sensitivity to ensure the use of an adequate quality control program. The combination of at least four colors is currently the best recommendation to monitor minimal residual disease in MM [39]. However, for most laboratories in developing countries, equipment that reads four colors simultaneously is still uncommon due to high costs. Discrepancies between cytological evaluation and FCM and their impact as predictors of relapse in MM could be attributed to the ability of FCM to detect malignant plasma cells as compared to BM cytology. 

 Our results could be attributed to a short followup, small sample size, and possibly, to the inability of a three-color parametric flow cytometry to correctly detect the malignant plasma cell population. We showed however that, in our hands, FCM is a high sensitive method for MRD detection. Augmenting the number of patients, the follow-up period, and the utilization of more then three colors combination for FCM will likely improve our results. The use of MRD burden for the early institution of treatment, as well as the use of novel drugs with a better remission quality will likely be widely utilized in the treatment of MM. 

## Figures and Tables

**Table 1:** Characteristics of MM patients enrolled in the study between December 2005 and May 2009.

Total patients (*n*)	44	
Age, years median (min–max)	55,5	52,2–64
Sex, M/F	21/23	
Immunoglobulin isotype (%)		
IgG	61,4	
IGA	18,20	
Kappa light chain	15,90	
Lambda light chain	4,5	
Response before TCTH *n* (% of total)		
CR	12	27,3
VGPR	19	43,2
PR	13	29,5
Response after TCTH *n* (% of total)		
CR	14	32,6
VGPR	16	36,4
PR	2	4,7
Not known	1	2,3
Relapse	3	7,0
Dead	8	18,6
Follow-up time, months (mean)	18	9–43

**Table 2:** Results of patients at 3 months.

	MRD (3 months)	Status (preTCTH)	Status (end of study)	Immunoglobulin type
1	−	VGPR	D	IgG
2	−	CR	CR	IgG
3	−	CR	CR	IgG
4	−	VGPR	D	IgG
5	−	CR	CR	Kappa light chain
6	−	PR	D	IgA
7	+	PR	VGPR	IgG
8	−	PR	VGPR	IgA
9	−	CR	CR	Kappa light chain
10	−	VGPR	R	Lambda light chain
11	+	PR	D	IgG
12	−	CR	VGPR	IgG
13	−	CR	CR	IgG
14	−	VGPR	PR	IgG
15	+	PR	D	IgA
16	+	PR	R	IgA
17	−	CR	∗	IgG
18	+	VGPR	CR	IgG
19	+	CR	VGPR	IgG
20	+	CR	CR	IgG
21	+	CR	CR	Lambda light chain
22	+	VGPR	VGPR	IgG
23	+	PR	VGPR	IgG
25	−	VGPR	VGPR	Kappa light chain
28	−	PR	D	IgG
30	−	VGPR	CR	IgG
31	−	CR	CR	IgG
32	−	VGPR	VGPR	IgG
33	−	CR	VGPR	IgG
35	+	PR	R	IgA
36	−	PR	VGPR	Kappa light chain
37	−	PR	D	IgA
38	+	VGPR	VGPR	IgG
39	+	VGPR	VGPR	Kappa light chain
40	+	VGPR	VGPR	IgA
41	+	PR	D	IgA
42	+	PR	PR	IgA
43	−	VGPR	VGPR	IgG
44	−	VGPR	VGPR	IgA
45	−	VGPR	VGPR	IgG

(D) Died; (R) relapse; (PR) partial remission; (VGPR) very good PR; (CR) complete remission.

(+) FCM > 0,01% neoplastic PC immunophenotype.

(−) FCM ≤ 0,01% PC neoplastic immunophenotype.
